# MRI Characterizes the Progressive Course of AD and Predicts Conversion to Alzheimer’s Dementia 24 Months Before Probable Diagnosis

**DOI:** 10.3389/fnagi.2018.00135

**Published:** 2018-05-24

**Authors:** Christian Salvatore, Antonio Cerasa, Isabella Castiglioni

**Affiliations:** ^1^Institute of Molecular Bioimaging and Physiology, National Research Council (IBFM-CNR), Milan, Italy; ^2^Institute of Molecular Bioimaging and Physiology, National Research Council (IBFM-CNR), Catanzaro, Italy

**Keywords:** artificial intelligence, Alzheimer’s disease, clinical trials, magnetic resonance imaging, neuropsychological tests, biomarkers, predictors

## Abstract

There is no disease-modifying treatment currently available for AD, one of the more impacting neurodegenerative diseases affecting more than 47.5 million people worldwide. The definition of new approaches for the design of proper clinical trials is highly demanded in order to achieve non-confounding results and assess more effective treatment. In this study, a cohort of 200 subjects was obtained from the Alzheimer’s Disease Neuroimaging Initiative. Subjects were followed-up for 24 months, and classified as AD (50), progressive-MCI to AD (50), stable-MCI (50), and cognitively normal (50). Structural T1-weighted MRI brain studies and neuropsychological measures of these subjects were used to train and optimize an artificial-intelligence classifier to distinguish mild-AD patients who need treatment (AD + pMCI) from subjects who do not need treatment (sMCI + CN). The classifier was able to distinguish between the two groups 24 months before AD definite diagnosis using a combination of MRI brain studies and specific neuropsychological measures, with 85% accuracy, 83% sensitivity, and 87% specificity. The combined-approach model outperformed the classification using MRI data alone (72% classification accuracy, 69% sensitivity, and 75% specificity). The patterns of morphological abnormalities localized in the temporal pole and medial-temporal cortex might be considered as biomarkers of clinical progression and evolution. These regions can be already observed 24 months before AD definite diagnosis. The best neuropsychological predictors mainly included measures of functional abilities, memory and learning, working memory, language, visuoconstructional reasoning, and complex attention, with a particular focus on some of the sub-scores of the FAQ and AVLT tests.

## Introduction

According to the World Health Organization, there were 47.5 million people worldwide with dementia in 2015, with 7.7 million new cases each year. The total number of people with dementia is projected to reach 75.6 millions in 2030 and almost triple by 2050 to 135.5 millions (Dementia Statistics, 2015; World Alzheimer Report, 2015; Khan et al., 2017). The most frequent dementia form is Alzheimer’s Disease (AD) (approximately 70%), whose impact on the society in terms of costs as well as quality of life of patients and families is impressive (Khan et al., 2017). There is no AD-modifying treatment available to date, and one third of the population will die with dementia if something does not change in the approach of screening, diagnosis, prognosis and treatment, including more proper design of clinical trials.

Currently, there are indeed more than 500 open clinical studies on AD, according to *ClinicalTrials.gov*. Many other clinical trials have been closed in the past years, few achieved phase III and no one demonstrated a proper success rate. Most of the past clinical trials enrolled people with advanced AD, and clinicians recommended to treat patients at an earlier stage for more effective results. Thus, current clinical trials try to enroll subjects at an early phase of the disease: inclusion criteria are now based on the selection of this specific patient group.

The patient’s self-reported experiences and the observed cognitive, functional and behavioral symptomatology due to AD over the longitudinal course of the illness are the current basis for the clinical diagnosis of AD. However, they are insufficient for detecting early AD subjects, considering also that only 33% of subjects with mild cognitive impairment (MCI) progress to AD (Mitchell and Shiri-Feshki, 2009). Furthermore, no standards have been defined on the best neuropsychological outcomes to be measured for this purpose.

For these reasons, clinical trials based only on neuropsychological assessment risk (1) including subjects with early dementia forms that are not caused by AD and (2) lasting several years prior to be completed, when most of the enrolled subjects have clearly progressed to AD. This leads to confounding clinical-trial designs, and cause treatments to be administered on patients who are not really affected by AD.

In 2011, after many scientific evidences, medical-imaging studies were included in the revised diagnostic criteria for AD in order to detect objective signs of disease in the subjects’ brain. Being positive to Positron Emission Tomography (PET) with Aβ- or tau-specific radiotracers is used as an inclusion criterion in most recent clinical trials, with the aim of measuring the presence of brain β-amyloid plaques or tau deposition, the recognized cause of AD pathogenesis. However, these PET studies are expensive, invasive and difficult to be implemented for technical and authorization problems, in particular in non-western countries. Moreover, lack of success in clinical trials of candidate drugs targeting amyloid or tau proteins has led to target alternative mechanisms (e.g., Khan et al., 2017).

Magnetic Resonance Imaging (MRI) is a less expensive technique than PET, non-invasive and more common in both western and non-western regions, and already recommended to detect AD neuronal degeneration and to monitor AD progression in clinical trials (Sperling et al., 2011). However, radiologists are not always able to detect -by visual inspection- the presence of subtle cerebral signs of neurodegeneration in MCI subjects, and even when this is possible, they are not able to predict if a subject will progress or not to AD.

Artificial-intelligence (AI) technology is emerging as an effective tool for automatic, objective and more sensitive assessment of imaging studies. Specifically, machine-learning (ML) and pattern-recognition techniques have captured the attention of the neuroimaging community as they have been proven able to discover previously unknown patterns in imaging data (Bishop, 2006; Wernick et al., 2010). In other words, these algorithms are able to (1) extract information from imaging data without *a priori* knowledge of where it may be encoded in the images, and (2) combine the information encoded in multiple inter- and intra-domain variables. This information can then be used to design multivariate mathematical models able to automatically predict the diagnostic class of a subject. This characteristic may be of particular usefulness in the context of early diagnosis, when pathological signs are not yet evident by visual inspection (Salvatore et al., 2015a). In the last years, different ML approaches have been applied to the automatic diagnosis and prognosis of AD by means of cerebral MRI studies, showing good performance even at an early stage of the disease (e.g., Cuingnet et al., 2011; Moradi et al., 2015; Salvatore et al., 2015b; Nanni et al., 2016). Furthermore, good results have been obtained to translate the hidden image features used by ML in performing subject classification, which are often typically complex features, counter-intuitive and not meaningful *per se* to clinicians (Haufe et al., 2014; Salvatore et al., 2015b; Huys et al., 2016). Thus, results of ML classification by means of MRI brain images can be more easily interpreted by clinicians and associated to AD pathogenesis.

The aim of this study is to refine the application of ML systems for the characterization of the progressive course of AD and to predict the conversion of MCI to AD, trying to establish how long before it would be possible to predict the diagnosis of probable AD. Application of this approach to longitudinal datasets would enable us to focus on the prognosis rather than the diagnosis and to identify cost-effective biomarkers, which may be targeted for prevention/intervention programs.

## Materials and Methods

### Participants

Data used in the preparation of this article were obtained from the Alzheimer’s Disease Neuroimaging Initiative (ADNI) database^1^. The ADNI was launched in 2003 by the National Institute on Aging (NIA), the National Institute of Biomedical Imaging and Bioengineering (NIBIB), and the Food and Drug Administration (FDA), as a 5-year public private partnership, led by the principal investigator, Michael W. Weiner, MD. The primary goal of ADNI was to test whether serial magnetic resonance imaging (MRI), positron emission tomography (PET), other biological markers, and clinical and neuropsychological assessments subjected to participants could be combined to measure the progression of MCI and early Alzheimer’s disease (AD) – see www.adni-info.org.

As specified in the ADNI protocol^2^, each participant was willing, spoke either English or Spanish, was able to perform all test procedures described in the protocol and had a study partner able to provide an independent evaluation of functioning.

Inclusion criteria for cognitively normal (CN) subjects were: Mini Mental State Examination (MMSE) (Folstein et al., 1975) scores between 24 and 30, Clinical Dementia Rating (CDR) of zero (Morris, 1993), and absence of depression, MCI and dementia. Inclusion criteria for MCI were: MMSE scores between 24 and 30, CDR of 0.5, objective memory loss measured by education-adjusted scores on the Logical Memory II subtest of the Wechsler Memory Scale (Wechsler, 1987), absence of significant levels of impairment in other cognitive domains, and absence of dementia. Inclusion criteria for AD were: MMSE scores between 20 and 26, CDR of 0.5 or 1.0, and criteria for probable AD as defined by the National Institute of Neurological and Communicative Disorders and Stroke (NINCDS) e by the Alzheimer’s Disease and Related Disorders Association (ADRDA) (McKhann et al., 1984; Dubois et al., 2007).

Serial MRI studies were performed to participants from baseline, covering a follow-up period of several years. Each participant was diagnosed at each time point of serial MRI studies.

In the present work, a total of 200 subjects were retrieved from the ADNI database, consisting into 50 subjects with a stable diagnosis of CN state over the 24 months of follow up, 50 subjects with a stable diagnosis of MCI (sMCI), 50 subjects with a stable diagnosis of AD, and 50 subjects with an initial diagnosis of MCI who showed a progression to AD (pMCI).

Two age- and sex-matched groups of subjects were created by grouping, separately, AD with pMCI (100 subjects) and CN with sMCI (100 subjects).

These subjects had all three serial MRI studies at three time points after the baseline: 6, 12, and 24 months.

The 24-months point was chosen as the time-zero point for a stable diagnosis. As a consequence, the three previous time points were reconsidered (and renamed) as *24 months before stable diagnosis*, *18 months before stable diagnosis*, and *12 months before stable diagnosis*.

Demographic and clinical characteristics of the groups of ADNI subjects considered in this study are shown in **Table 1**. ADNI *Subject IDs* as well as *Image Data IDs* can be found at the following online repository: https://github.com/christiansalvatore/Salvatore-200Longitudinal.

**Table 1:** Demographic and clinical characteristics of the subjects considered in this study.

Group type (stable diagnosis)	# Subjects	Age mean ± std. [range]	Gender #M/#F (%)
CN or sMCI	100	74.8 ± 6.4 [58.0–87.7]	55%
pMCI or AD	100	74.7 ± 7.1 [55.3–88.4]	54%


### MRI and Neuropsychological Data

For each subject of **Table 1**, and for each time point (*24 months before stable diagnosis*, *18 months before stable diagnosis*, *12 months before stable diagnosis*, and *time-zero point of stable diagnosis*), structural MR images were downloaded from the ADNI data repository. According to the ADNI acquisition protocol (Jack et al., 2008), examinations were performed at 1.5 T using a T1-weighted sequence. We considered MR images that had undergone the following preprocessing steps: (1) 3D gradwarp correction for geometry correction caused by gradient non-linearity (Jovicich et al., 2006), and (2) B1 non-uniformity correction for intensity correction caused by non-uniformity (Narayana et al., 1988). These preprocessing steps help improving the standardization among MR images from different MR sites and different platforms. MR images were downloaded in 3D NIfTI format. A further processing procedure was then performed on the downloaded images, this procedure consisting in: (1) image re-orientation; (2) cropping; (3) skull-stripping; (4) image normalization to the MNI standard space by means of co-registration to the MNI template (MNI152 T1 1 mm brain) (Grabner et al., 2006; O’Hanlon et al., 2013). MR images were then segmented into Gray Matter (GM) and White Matter (WM) tissue probability maps, and smoothed using an isotropic Gaussian kernel with Full Width at Half Maximum (FWHM) ranging from 2 to 12 mm^3,^ with a step of 2 mm^3^. After this phase, all MR images (whole-brain, GM and WM) resulted to be of size 121 × 145 × 121 voxels. The whole process was performed using the VMB8 software package installed on the Matlab platform (Matlab R2016b, The MathWorks). MRI volumes were visually inspected for checking homogeneity and absence of artifacts both before and after the pre-processing step.

Neuropsychological data were also obtained for each subject and for each time point from the ADNI data repository. Neuropsychological data included both scores and subscores of seven neuropsychological tests, namely the Functional Assessment Questionnaire (FAQ), the Clock Test, the Rey Auditory Verbal Learning Test (AVLT), the Digit Span (DS), the Category Fluency Tests (Animals and Vegetables), the Trail Making Test A-B (TMT A-B), and the Boston Naming Test (BNT). The full list of neuropsychological scores and subscores used in this study is reported in the Supplementary Table S1. All scores and subscores underwent a *z*-score normalization before being fed into the classification algorithm.

### The Classification

For each subject of **Table 1**, and for each time point, T1-weighted structural MR images and neuropsychological scores (and sub-scores) were used as input data of an automatic binary classifier to discriminate the two groups of subjects: (*CN* + *sMCI*) vs. (*pMCI* + *AD*).

For this purpose we used an AI system based on a supervised ML algorithm, tailored to learn from MRI images the prediction model to classify different diagnostic AD groups (Salvatore et al., 2015b).

The whole procedure is detailed in the following Sub-sections and consists into: extraction of features from the three different segmented MR images (whole-brain, GM or WM); ranking of features extracted from MR images; ranking of normalized neuropsychological scores and sub-scores; classification of subjects using the extracted and ranked features, further selected according to their ranking through a wrapper procedure. This procedure is repeated for different combinations of selected features, and the classifier is optimized on that combination showing the best classification performance (wrapper feature selection and optimization of classification).

#### Feature Extraction and Ranking

Feature extraction and feature ranking were performed to reduce the number of features to be handled by the classification algorithm, to remove the noisy features while keeping the ones relevant for group discrimination, and to reduce redundancy in the dataset. Thus, this step allowed an enhancement of the performance of the ML classifier while reducing computational costs.

A Principal Component Analysis (PCA) was implemented to perform feature extraction from the MRI volumes (López et al., 2011; Salvatore et al., 2015a). In particular, this method consists in applying and orthogonal transformation to the original set of variables in order to obtain a new (smaller) set of orthogonal variables called principal components. These new variables define a subspace, called the PCA subspace. The original dataset is then projected onto the PCA subspace, this operation resulting in a smaller set of features which are referred to as PCA coefficients and which can be used to replace the original dataset. This new dataset of PCA coefficients maximizes the variance of the dataset, under the constraint of orthogonality among the extracted variables. The number of extracted features cannot be higher than the value of the smaller dimension of the original dataset – 1. In our case, being the dimension of the dataset equal to *S* × *N*, where *S* is the number of samples (200) and *N* the number of features (MRI voxels + neuropsychological features, > 10^6^), then the number of extracted PCA coefficients will be at most 199.

Feature ranking was applied to PCA coefficients extracted from MR images, as well as to neuropsychological scores and sub-scores. FDR was implemented to perform feature ranking, which aims at sorting features according to their class-discriminatory power. This index was computed for each variable as follows:

$$FDR=\frac{{\left(\mu_{A}-\mu_{B}\right)}^{2}}{\sigma_{A}^{2}+\sigma_{B}^{2}}$$

where the numerator expresses the squared difference between the mean of that variable in class A and class B, while the denominator expresses the sum of the squared variances of that variable in class A and in class B.

A second independent feature-extraction technique based on Partial Least Squares (PLS) (Wold et al., 1984; Ramírez et al., 2010; Khedher et al., 2015) was implemented. The approach used in PLS is similar to the one used in PCA. However, differently from PCA, this technique involves the concurrent use of information from both the set *X* of observed variables (the original dataset itself) and the corresponding set *T* of diagnostic labels. Specifically, PLS consists in computing orthogonal vectors (also in this case called components) by maximizing the covariance between the two sets of variables *X* and *T*. The original variables are then projected onto the new space spanned by the computed orthogonal vectors. These projections are then used as input features for the classification system.

The feature-extraction-and-ranking technique based on PCA+FDR and the feature-extraction technique based on PLS were implemented independently from each other. The performances of the classifier implemented using these two techniques were then compared.

#### The Classifier

A Support Vector Machine (SVM) was used as a binary classifier (Cortes and Vapnik, 1995). The SVM algorithm was able to construct a predictive model based on a set of features from subjects with known stable diagnosis, called training dataset. This predictive model was then used to automatically classify new subjects (with unknown diagnosis) as belonging to one of the two diagnostic classes.

The predictive model computed by SVM was the one that maximized the margin between the two diagnostic classes, represented by a hyper-plane whose analytical form is given by:

$$y(x)=\overset{N}{\underset{n=1}{\Sigma }}w_{n}•t_{n}•k(x,x_{n})+b$$

Here *N* is the number of subjects in the training set, *w*_n_ is the weight assigned by SVM to each subject n in the training set during the training phase, *t*_n_ represents the diagnosis of the subject *n* of the training set, *k*(*x*,*x*_n_) is the kernel function, and b is a threshold parameter.

In our analyses, we implemented a linear kernel SVM on the Matlab platform (R2016b, The MathWorks), also including algorithms from the biolearning toolbox of Matlab.

#### Wrapper Feature Selection, Optimization of Classification, Performance Evaluation

In order to find the best configuration of parameters for the classification, a wrapper feature selection and optimization of classification was performed. Specifically, the features to be selected were the MRI features extracted and ranked using PCA and FDR, and the neuropsychological scores and sub-scores normalized and ranked using FDR. The parameters to be optimized were only related to the MR image preprocessing, and they included the tissue probability map (whole-brain, GM or WM), and the FWHM of the smoothing kernel (FWHM = 2, 4, 6, 8, 10, and 12 mm^3^ or no smoothing).

Wrapper feature selection and optimization were performed using a fivefold Nested-Cross-Validation (Nested CV) approach (Varma and Simon, 2006). In this approach, the original dataset (100 subjects with CN or sMCI and 100 subjects with AD or pMCI) was split into 5 subsets of equal size: 4/5 subsets were used in an inner training-and-validation loop to perform feature selection and parameter optimization; the remaining 1/5 subset was then used in an outer test loop for the performance evaluation of the classifier. This procedure was repeated five times, until all subsets were used once for testing in the outer loop.

For each round, the set of selected features and optimal parameters was estimated in the inner loop as the one that maximized the accuracy of classification. For each round, the performance was estimated in the outer loop in terms of accuracy, sensitivity, and specificity of classification. Mean accuracy, sensitivity and specificity was calculated averaging across all 5 rounds.

Given that the number of subjects in the whole dataset was 200 (i.e., 100 CN + sMCI and 100 pMCI + AD), for each round of nested CV the number of subjects used to train the classifier was 128, the number of subjects used to optimize the classifier was 32 (inner loop), and the number of subjects used to evaluate the performance of the classifier was 40 (outer loop).

The whole process was performed for each time point (*24 months before stable diagnosis*, *18 months before stable diagnosis*, and *12 months before stable diagnosis*).

In order to assess the statistical significance of each performance metric (accuracy, sensitivity, and specificity of classification), we performed a permutation test. Specifically, the classifier was run as described above, but the labels were computed as a random permutation of the original label set. This procedure was repeated for a total of 1000 iterations. A *p*-value indicating the statistical significance of each performance metric was then calculated as the fraction of the total number of iterations for which the performance (accuracy, sensitivity, or specificity, respectively) resulted to be greater than or equal to the performance observed using the original labels.

### MRI and Neuropsychological Predictors

A three-dimensional map of voxel-based intensity distribution of MRI differences between (CN + sMCI) and (pMCI + AD) was generated for each round of the inner training-and-validation loop. The map was created for the set of selected features and optimal parameters obtained using the PCA+FDR feature-extraction-and-ranking technique. The maps generated during the 5 rounds of nested CV were then averaged in a single final map.

The importance of each voxel was computed as in our previous papers (Cerasa et al., 2015; Salvatore et al., 2015b) based on the predictive model generated by SVM. Specifically, during the training phase, SVM assigns a weight to each sample in the training set corresponding to the importance of that sample in defining the predictive model. By multiplying each sample of the training set by the corresponding weight, and by adding resulting weighted samples on a voxel-basis, it is possible to generate a three-dimensional map of the weights of each voxel. Furthermore, the method proposed by Haufe et al. (2014) to compute activation patterns in backward models was applied in order to ensure the correct interpretation of the weights.

Voxel-based maps were then normalized in intensity (to a range between 0 and 1) and superimposed on a standard stereotactic brain using a proper color scale. This procedure was performed for each time point (*24 months before stable diagnosis*, *18 months before stable diagnosis*, and *12 months before stable diagnosis*) (Cerasa et al., 2015; Salvatore et al., 2015b).

The most frequent neuropsychological scores and subscores among those selected in all rounds were also identified. Also in this case, these results were obtained for the classifier implemented using the PCA+FDR feature-extraction-and-ranking technique. These features were sorted in descending order according to their frequency. The features occurring with a higher frequency than 5% were shown as best predictors.

## Results

### The Classification

Classification results when using PCA+FDR as feature-extraction-and-ranking technique are shown in **Table 2** for the classification of (*CN* + *sMCI*) vs. (*pMCI* + *AD*). Using only MRI data, accuracy, sensitivity, and specificity of the classification were 0.72 ± 0.08, 0.69 ± 0.12, and 0.75 ± 0.08, respectively, at the time point *24 months before stable diagnosis;* 0.77 ± 0.05, 0.78 ± 0.07, and 0.76 ± 0.10 at the time point *18 months before stable diagnosis;* 0.75 ± 0.08, 0.79 ± 0.14, and 0.71 ± 0.11 at the time point *12 months before stable diagnosis*. As a benchmark, we also measured the performance of the classifier in discriminating (CN + sMCI) vs. (pMCI + AD) at the time-zero point of stable diagnosis (that is, when all pMCI had manifested their progression to AD). In this case, accuracy, sensitivity and specificity resulted to be 0.79 ± 0.08, 0.83 ± 0.14, and 0.75 ± 0.10, respectively. The performances of the proposed method result to be statistically significant as assessed by means of permutation tests (*p* < 0.001). On the other side, no statistical difference was found among the performance obtained at the four different time points (*p* = 0.51, one-way ANOVA). The *p*-values (multiple comparisons for one-way ANOVA) for all the possible binary combinations of time points are reported in the Supplementary Table S2.

**Table 2:** Classification performance in terms of accuracy, sensitivity, and specificity for (CN + sMCI) vs. (pMCI + AD) at the considered time points, using MR images alone or coupled with neuropsychological measures, with PCA+FDR as feature-extraction-and-ranking technique.

	24 m before stable diagnosis	18 m before stable diagnosis	12 m before stable diagnosis	Stable-diagnosis time point
MRI		
Accuracy	0.72 ± 0.08	0.77 ± 0.05	0.75 ± 0.08	0.79 ± 0.08
Sensitivity	0.69 ± 0.12	0.78 ± 0.07	0.79 ± 0.14	0.83 ± 0.14
Specificity	0.75 ± 0.08	0.76 ± 0.10	0.71 ± 0.11	0.75 ± 0.10
MRI + Neuropsychological data		
Accuracy	0.85 ± 0.05	0.85 ± 0.09	0.87 ± 0.06	0.92 ± 0.01
Sensitivity	0.83 ± 0.09	0.86 ± 0.11	0.86 ± 0.11	0.91 ± 0.04
Specificity	0.87 ± 0.06	0.83 ± 0.17	0.87 ± 0.03	0.93 ± 0.03


**The performance of the classifier at the time point of the stable diagnosis is also shown.**

When using MRI and neuropsychological data in combination, accuracy, sensitivity, and specificity were 0.85 ± 0.05, 0.83 ± 0.09, and 0.87 ± 0.06, respectively, at the time point *24 months before stable diagnosis;* 0.85 ± 0.09, 0.86 ± 0.11, and 0.83 ± 0.17 at the time point *18 months before stable diagnosis;* 0.87 ± 0.06, 0.86 ± 0.11, and 0.87 ± 0.03 at the time point *12 months before stable diagnosis*. Accuracy, sensitivity and specificity at the time-zero point of stable diagnosis were 0.92 ± 0.01, 0.91 ± 0.04, and 0.93 ± 0.03, respectively. The performances of the proposed method result to be statistically significant as assessed by means of permutation tests (*p* < 0.001). On the other side, no statistical difference was found among the performance obtained at the four different time points (*p* = 0.20, one-way ANOVA). The *p*-values (multiple comparisons for one-way ANOVA) for all the possible binary combinations of time points are reported in the Supplementary Table S3.

Furthermore, when comparing –at different time points– the accuracy of classification obtained using MRI and neuropsychological data in combination with respect to the one obtained using MRI alone, the combined approach resulted to perform statistically better -at the 5% significance level- than the single-modality approach at the time points of 24 months before stable diagnosis (*p* = 0.01), 12 months before stable diagnosis (*p* = 0.03), and at the stable-diagnosis time point (*p* = 0.01). No statistical difference was found at the time point of 18 months before stable diagnosis (*p* = 0.15).

Classification results obtained when using PLS as feature extraction technique are shown in **Table 3**. Using only MRI data, accuracy, sensitivity and specificity of the classification were 0.79 ± 0.07, 0.79 ± 0.07, and 0.78 ± 0.08, respectively, at the time point *24 months before stable diagnosis;* 0.81 ± 0.04, 0.81 ± 0.07, and 0.81 ± 0.07 at the time point *18 months before stable diagnosis;* 0.81 ± 0.05, 0.83 ± 0.08, and 0.79 ± 0.05 at the time point *12 months before stable diagnosis*. The benchmark performance of the classifier at the time-zero point of stable diagnosis was 0.82 ± 0.04 accuracy, 0.82 ± 0.07 sensitivity and 0.81 ± 0.04 specificity. The performances of the proposed method resulted to be statistically significant as assessed by means of permutation tests (*p* < 0.001). No statistical difference was found among the performance obtained at the four different time points (*p* = 0.76 for accuracy, one-way ANOVA). The *p*-values (multiple comparisons for one-way ANOVA) for all the possible binary combinations of time points are reported in the Supplementary Table S4.

**Table 3:** Classification performance in terms of accuracy, sensitivity, and specificity for (CN + sMCI) vs. (pMCI + AD) at the considered time points, using MR images alone or coupled with neuropsychological measures, with PLS as feature-extraction technique.

	24 m before stable diagnosis	18 m before stable diagnosis	12 m before stable diagnosis	Stable-diagnosis time point
MRI		
Accuracy	0.79 ± 0.07	0.81 ± 0.04	0.81 ± 0.05	0.82 ± 0.04
Sensitivity	0.79 ± 0.07	0.81 ± 0.07	0.83 ± 0.08	0.82 ± 0.07
Specificity	0.78 ± 0.08	0.81 ± 0.07	0.79 ± 0.05	0.81 ± 0.04
MRI + Neuropsychological data		
Accuracy	0.81 ± 0.07	0.83 ± 0.12	0.84 ± 0.06	0.85 ± 0.05
Sensitivity	0.82 ± 0.08	0.83 ± 0.10	0.86 ± 0.07	0.87 ± 0.09
Specificity	0.80 ± 0.11	0.83 ± 0.18	0.82 ± 0.10	0.83 ± 0.04


**The performance of the classifier at the time point of the stable diagnosis is also shown.**

When using a combination of MRI and neuropsychological data, accuracy, sensitivity and specificity were 0.81 ± 0.07, 0.82 ± 0.08, and 0.80 ± 0.11, respectively, at the time point *24 months before stable diagnosis;* 0.83 ± 0.12, 0.83 ± 0.10, and 0.83 ± 0.18 at the time point *18 months before stable diagnosis;* 0.84 ± 0.06, 0.86 ± 0.07, and 0.82 ± 0.10 at the time point *12 months before stable diagnosis*. The benchmark performance of the classifier in terms of accuracy, sensitivity and specificity at the time-zero point of stable diagnosis was 0.85 ± 0.05, 0.87 ± 0.09, and 0.83 ± 0.04, respectively. The performances of the proposed method result to be statistically significant as assessed by means of permutation tests (*p* < 0.001). No statistical difference was found among the performance obtained at the four different time points (*p* = 0.88 for accuracy, one-way ANOVA). The *p*-values (multiple comparisons for one-way ANOVA) for all the possible binary combinations of time points are reported in the Supplementary Table S5.

Furthermore, when comparing –at different time points– the accuracy of classification obtained using MRI and neuropsychological data in combination with respect to the one obtained using MRI alone, no statistical difference was observed (*p* = 0.23 at the time point of 24 months before stable diagnosis; *p* = 0.65 at the time point of 18 months before stable diagnosis; *p* = 0.11 at the time point of 12 months before stable diagnosis; *p* = 0.08 at the stable-diagnosis time point).

Making a pairwise comparison (paired-sample *t*-test) between the performance obtained using PCA+FDR vs. PLS (for each time point and for each domain), results show that -at the 5% significance level- the classifier implemented using PLS performed statistically better (in terms of accuracy) than the one implemented using PCA+FDR at the time points of 24 and 18 months before stable diagnosis when using MRI alone (*p* = 0.03 in both cases). A comprehensive table showing all pairwise *p*-values can be found in Supplementary Table S6.

### MRI and Neuropsychological Predictors

**FIGURE 1:**
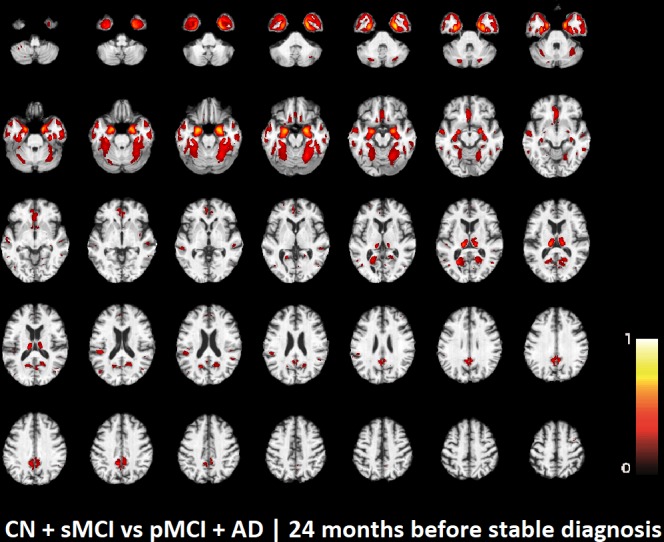
Voxel-based pattern distribution of MRI differences between CN + sMCI and pMCI + AD at the time point 24 months before stable diagnosis. The pattern is shown according to the color scale with a threshold of 35%, and superimposed on a standard stereotactic brain.

**FIGURE 2:**
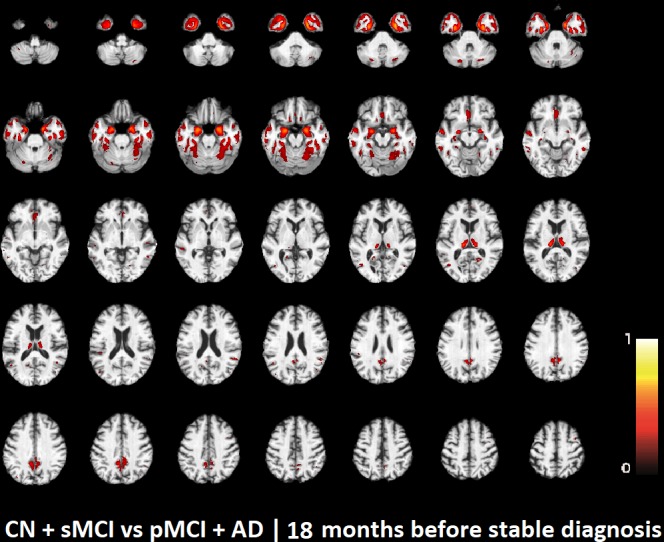
Voxel-based pattern distribution of MRI differences between CN + sMCI and pMCI + AD at the time point 18 months before stable diagnosis. The pattern is shown according to the color scale with a threshold of 35%, and superimposed on a standard stereotactic brain.

**FIGURE 3:**
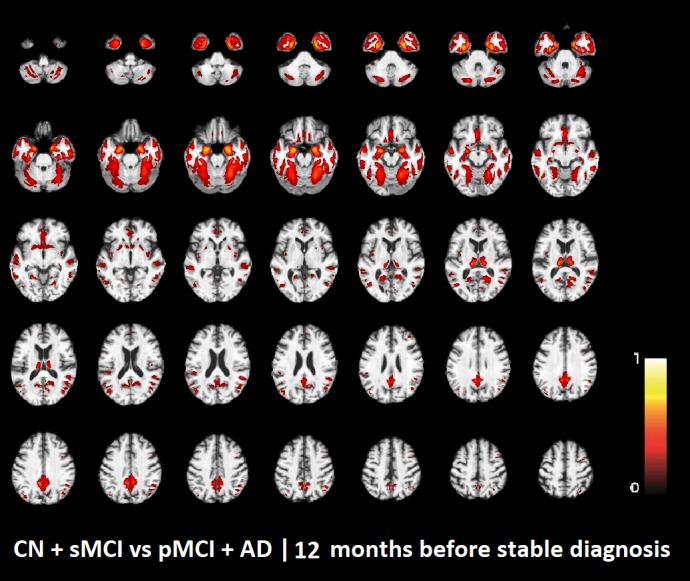
Voxel-based pattern distribution of MRI differences between CN + sMCI and pMCI + AD at the time point 12 months before stable diagnosis. The pattern is shown according to the color scale with a threshold of 35%, and superimposed on a standard stereotactic brain.

**FIGURE 4:**
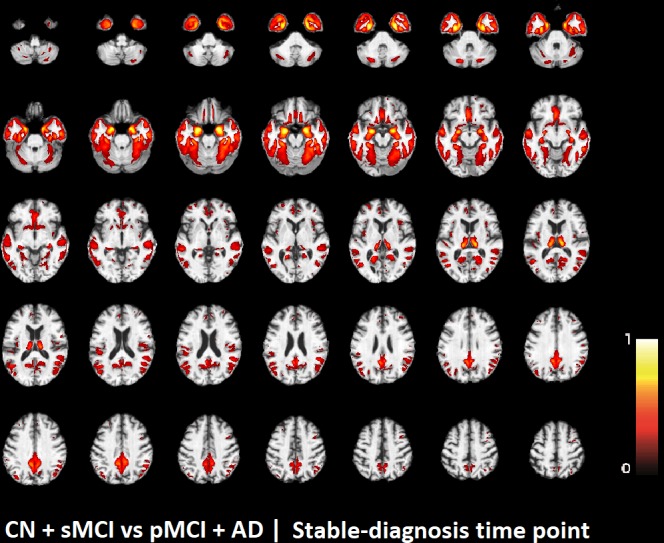
Voxel-based pattern distribution of MRI differences between CN + sMCI and pMCI + AD at the time-zero point of stable diagnosis. The pattern is shown according to the color scale with a threshold of 35%, and superimposed on a standard stereotactic brain.

The voxel-based pattern distribution of MRI differences found as results of classification between CN + sMCI and pMCI + AD are shown in **Figures 1**–**3**, for the three considered time points, respectively (i.e., *24 months before stable diagnosis*, *18 months before stable diagnosis*, and *12 months before stable diagnosis*). The voxel-based pattern distribution of MRI differences at the time-zero point of stable diagnosis is also shown in **Figure 4**. All patterns were shown according to the color scale with a threshold of 35%, and superimposed on a standard stereotactic brain in order to allow a better localization of the brain regions identified by the classifier.

Similarly, the best neuropsychological predictors and corresponding status/domain/subdomain found for the classification of (CN + sMCI) vs. (pMCI + AD) for the considered time-points are reported in **Table 4**. Findings are sorted in descending order according to their frequency. The complete list of best neuropsychological predictors with the corresponding names as reported in the ADNI data repository can be found in Supplementary Table S7.

**Table 4:** Best Neuropsychological predictors and corresponding status/domain/subdomain found for the classification of (CN + sMCI) vs. (pMCI + AD).

Time point	Neuropsychological predictor	Status/domain/subdomain of predictor
*24 months before stable diagnosis*	Ability in remembering appointments, family occasions, holidays, medications in FAQ	Functional abilities
	Ability in writing checks, paying bills, or balancing checkbook in FAQ	Functional abilities
	Ability in assembling tax records, business affairs in FAQ	Functional abilities
	Total score of trial 5 in AVLT	Memory and learning
	Ability in keeping track of current events in FAQ	Functional abilities
	Total intrusions of trial 1 in AVLT	Memory and learning
	Correct answers in the Backwards task in Digit-Span Test	Working memory
	Correct answers in Vegetables task in Category Fluency Test	Language
	Correct answers after a 30-min delay in AVLT	Memory and learning
*18 months before stable diagnosis*	Ability in writing checks, paying bills, or balancing checkbook in FAQ	Functional abilities
	Ability in remembering appointments, family occasions, holidays, medications in FAQ	Functional abilities
	Total score of trial 3 in AVLT	Memory and learning
	Total score of trial 5 in AVLT	Memory and learning
	Total score of trial 6 in AVLT	Memory and learning
	Ability in assembling tax records, business affairs in FAQ	Functional abilities
	Ability in traveling, driving, or arranging to take public transportation in FAQ	Functional abilities
	Presence of the two hands in CLOCK test	Visuoconstructional reasoning
	Ability in shopping alone for necessities in FAQ	Functional abilities
	Ability in keeping track of current events in FAQ	Functional abilities
	Total score of FAQ	Functional abilities
	Total of trial 4 in AVLT	Memory and learning
	Spontaneously given correct responses in BNT	Language
	Corrected responses following phonemic cues in BNT	Language
	Symmetry of number placement in CLOCK test	Visuoconstructional reasoning
	Presence of the two hands, set to ten after eleven in CLOCK test	Visuoconstructional reasoning
	Time to complete Part A of the test in TMT	Complex attention
	Time to complete Part B of the test in TMT	Complex attention
	Correct answers after a 30-min delay in AVLT	Recognition errors in AVLT
	Memory and learning	Memory and learning
*12 months before stable diagnosis*	Ability in writing checks, paying bills, or balancing checkbook in FAQ	Functional abilities
	Ability in remembering appointments, family occasions, holidays, medications in FAQ	Functional abilities
	Total of trial 3 in AVLT	Memory and learning
	Number of correct responses following a phonemic cue in BNT	Language
	Ability in assembling tax records, business affairs in FAQ	Functional abilities
	Ability in shopping alone for necessities in FAQ	Functional abilities
	Ability in traveling, driving, or arranging to take public transportation in FAQ	Functional abilities
	Total score of FAQ	Functional abilities
	Total of trial 4 in AVLT	Memory and learning
	Total of trial 5 in AVLT	Memory and learning
	Total correct answers after a 30-min delay in AVLT	Memory and learning
	Total of trial 6 in AVLT	Memory and learning
	Ability in keeping track of current events in FAQ	Functional abilities
	Ability in paying attention to and understanding a TV program, book, or magazine in FAQ	Total score of the CLOCK test
	Functional abilities	Visuoconstructional reasoning
*Time-zero point of stable diagnosis*	Ability in writing checks, paying bills, or balancing checkbook in FAQ	Functional abilities
	Total score of FAQ.	Functional abilities
	Total of trial 4 in AVLT	Memory and learning
	Ability in remembering appointments, family occasions, holidays, medications in FAQ	Functional abilities
	Ability in paying attention to and understanding a TV program, book, or magazine in FAQ	Functional abilities
	Ability in traveling out of the neighborhood, driving, arranging to take public transportation in FAQ	Functional abilities
	Ability in assembling tax records, business affairs, or other papers in FAQ	Functional abilities
	Ability of the subject in preparing a balanced meal in FAQ	Functional abilities
	Total of trial 6 in AVLT	Memory and learning
	Ability in playing a game of skill such as bridge or chess, working on a hobby in FAQ	Correct answers after a 30-min delay in AVLT
	Functional abilities	Memory and learning


**Results are reported for the three considered time points (i.e., 24 months before stable diagnosis, 18 months before stable diagnosis, and 12 months before stable diagnosis) and for the time-zero point of stable diagnosis. Best neuropsychological predictors are sorted in descending order according to their frequency (the frequency of that measure in all loops). The status/domain/subdomain corresponding to the neuropsychological predictor is also reported*.*

## Discussion

The main finding of our work was that, using structural T1-weighted MRI brain studies and specific neuropsychological measures, our classifier was able to identify mild-AD patients who need treatments 24 months before AD definite diagnosis with an 85% accuracy, 83% sensitivity, and 87% specificity (see **Table 2**, when considering the method implemented using PCA+FDR). More interestingly, the performance obtained by our multi-modal classifier in distinguishing normal subjects (or stable MCI) from patients who will evolve to AD 24 months before stable diagnosis is comparable (*p* > 0.2) to the ones obtained at 18, 12 months before stable diagnosis and, even more important, to the one obtained at the time of definite diagnosis. Furthermore, the combined classification approach model outperformed the other classification considered in this study using single MRI data (72% classification accuracy, 69% sensitivity, and 75% specificity) (**Table 2**, *p* < 0.05, when considering the method implemented using PCA+FDR).

Although the discrimination of (CN + sMCI) vs. (pMCI + AD) is not common in the literature, our results can be compared with the classification performance of studies focused on predicting the conversion to Alzheimer’s dementia. These studies usually limit their attention to the binary classification of *pMCI* vs. *sMCI*. In a recent review considering 30 studies applying ML for the diagnosis of AD using only structural MRI (Salvatore et al., 2015a), the mean classification accuracy in discriminating *pMCI* vs. *sMCI* was found to be 0.66 ± 0.11. Another study tried to distinguish AD patients from stable MCI patients using only structural MRI features (Diciotti et al., 2012). A classification accuracy of 0.74 was reported (0.72 sensitivity, 0.77 specificity), although they used a private cohort of 21 mild AD and 30 MCI patients, and the gold-standard diagnosis was not based on follow-up examinations. Some other studies tried to automatically classify *pMCI* vs. *sMCI* using only MRI features (e.g., Cui et al., 2011; Koikkalainen et al., 2012; Ye et al., 2012; Casanova et al., 2013; Peters et al., 2014; Runtti et al., 2014; Dukart et al., 2015; Eskildsen et al., 2015; Moradi et al., 2015; Ritter et al., 2015; Salvatore et al., 2015b; Nanni et al., 2016), with a classification accuracy ranging from 0.51 to 0.75.

To the best of our knowledge, this is one of the few works able to answer the question whether a multidisciplinary classification model coupling cognitive, functional and behavioral measures with structural MRI brain studies is better than a model based only on structural MRI. Four studies attempted the task of classifying *pMCI* vs. *sMCI* using both structural-MRI features alone and in combination with neuropsychological measures (Cui et al., 2011; Runtti et al., 2014; Dukart et al., 2015; Moradi et al., 2015). The classification accuracy of these studies ranges from 0.62 to 0.75 when using structural MRIs alone, and from 0.62 to 0.82 when using both structural MRIs and neuropsychological measures, showing a slight improvement (the mean intra-study improvement was 0.06 ± 0.04).

Another challenging finding of our study was that patterns of morphological abnormalities localized in the temporal pole and medial-temporal cortex might be considered as biomarkers of clinical progression and evolution (**Figures 1**–**4**). These regions can be already observed at the time point of *24 months before stable diagnosis* (**Figure 1**). When considering the subsequent time points (**Figures 2**–**4**), the voxel-based pattern distribution of MRI-related neurodegeneration is similar to that one at 24 months before stable diagnosis, but progressively more extended, which could be a consequence of a more advanced process of structural neurodegeneration. There is an increasing interest proven by literature in understanding progression-related brain changes using structural MRI, describing an association between progression and atrophy, especially of the parietal and posterior cingulate regions, extending into the precuneus and medial temporal regions including hippocampus, amygdala, and entorhinal cortex. This pattern of progression-atrophy association is even evident at mild stages of cognitive impairment. The purpose of our work is out from explaining mechanisms behind the structural pattern distribution related to MRI images of different stages of disease progression. However, the progressive pattern seems to be consistent with Braak pathological studies (Braak and Braak, 1991), showing that during the development of AD pathology, tau tangles increase, associated with synapse loss and neurodegeneration.

Finally, we demonstrated that some cognitive, functional, and behavioral measures emerged as best predictors for AD progression. These include measures of functional abilities, memory and learning, working memory, language, visuoconstructional reasoning, and complex attention (see **Table 4**). More specifically, the best neuropsychological predictors for the classification of (*CN* + *sMCI*) vs. (*pMCI* + *AD*) at the time point of *24 months before stable diagnosis* include measures of functional abilities, memory and learning, working memory, and language. When considering the subsequent time points, involved domains are similar to the ones at 24 months before stable diagnosis. Interestingly, some of the sub-scores obtained through the administration of the FAQ (domain: functional abilities) and AVLT (domain: memory and learning) are always selected as best neuropsychological predictors at all the considered time points. Moreover, it must be noted that the best neuropsychological predictors at the time point of *stable diagnosis* include only measures from these two tests, which could be a consequence of a more advanced impairment in these two domains. Neuropsychological assessment can be time intensive, and the experience of practitioners can impact on the reliability and efficiency of the assessment. Our results can help the work of clinicians in optimizing the choice of cognitive tests to be administered at no costs for effectiveness. In a previous study of our group, Battista et al. (2017) demonstrated that it is possible to use a selected subset of neuropsychological measures to automatically diagnose AD patients with an accuracy of 90%.

It should be underlined that -in the present study- most of the best neuropsychological predictors at the time point of *24 months before stable diagnosis* are components of the AVLT or partial scores of FAQ related to learning and verbal episodic memory or prospective memory. These findings may confirm that the best neuropsychological predictors of conversion from amnestic MCI to AD are tests of episodic memory, as recently pointed out by Gainotti et al. (2014). Furthermore, also in the above-cited paper by Battista et al. (2017) the subset of selected neuropsychological measures able to automatically diagnose AD patients was mainly composed of measures related to episodic memory (namely, scores and subscores of AVLT, Logical Memory Test and Alzheimer’s Disease Assessment Scale-Cognitive Behavior) and measures addressing functional abilities in daily life (namely, total score and subscores of FAQ).

With respect to the numerous other ML methods proposed for the automatic classification of AD patients by means of brain MRI images (Cuingnet et al., 2011; Salvatore et al., 2015a), our approach has several points of strength.

Firstly, we validated our data on a large, multi-center independent cohort study, namely the ADNI public database. The use of large, public cohorts for training machine-learning classifiers allows a higher generalization ability than using private cohorts, which are often obtained from single-center studies. Moreover, the use of public databases is crucial for the comparison of the classification performance of different studies (Cuingnet et al., 2011), which is not recommended for studies using different private inhomogeneous cohorts. Mainly because of these reasons, in the last few years, the use of large, public data repositories is becoming more frequent in the field of ML applied to neuroimaging data, as reported in a recent review (Salvatore et al., 2015a). However, to date this is not a standard practice, and several studies still make use of private cohorts.

A second point of strength is that our algorithm requires a limited number of imaging studies to be trained, nearly a hundred studies per diagnostic class. This point is particularly important if considered with respect to the new classification approaches that are recently emerging as state-of-the-art techniques in the computer-vision community, namely deep-learning. These techniques have proven to be high performing in most automatic-classification tasks (Sharif Razavian et al., 2014), but their application in medicine, in particular in the neuroimaging field, is still limited. This is due to the requirement of at least a thousand of imaging studies per diagnostic class in order to reduce overfitting problems.

The third point of strength is the ability of our classification algorithm to return the best MRI and neuropsychological predictors, that is, the most important structural-brain patterns and neuropsychological scores for distinguishing the two diagnostic classes. Specifically, these predictors can be interpreted as early signs of the disease, and thus be used as surrogate biomarkers of AD. In the case of structural-MRI predictors, this may be particularly useful in monitoring the course of the neurodegeneration or the efficacy of a treatment.

Another advantage of our classification algorithm is that data used as input can be collected in a single examination session following routinely clinical protocols (T1-weighted MRI on 1.5T systems) and non-invasive and inexpensive measures obtained through the administration of standard neuropsychological tests.

Lastly, with respect to the use of structural MRI volumes, it must be noted that our classification algorithm does not require any interaction or pre-processing by the neuroradiologists on the original acquired images. This helps avoiding any issue arising from inter- and intra-operator inhomogeneities.

From a methodological point of view, we must underline two further points of strength. The first is the number of features used for training the classification algorithm, which was lower than the number of subjects in the two classes. This practice is useful as it prevents any curse-of-dimensionality issue. The second is the independence between neuropsychological measures used as features and measures used as gold standard to perform the original classification in the four diagnostic groups (AD, pMCI, sMCI, and CN). This practice warrants the avoidance of double-dipping in the classification process (Kriegeskorte et al., 2009).

However, we should also recognize some limitations of our work:

*Limited Generalization Ability and Reliability*. Further investigations are needed in order to assess the generalization ability and reliability of our multimodal MRI/cognitive-based classifier, and its applicability at an individual subject level. Our results are based on subjects in the United States and Canada, thus validation studies including subjects from other regions worldwide are lacking. Moreover, our predictive results have been obtained by a cross-validation approach using these subjects, and this may not accurately generalize our findings to a general population. We have used an SVM classifier since it offers different advantages, for example, is particularly appropriate for non-linear and big data such as whole-brain MRI images, also in combination with data from other modalities (e.g., biological and neuropsychological data). However, in order to confirm our results, we should have used more classifiers among the variety of ML methods already validated for automatic classification of medical images, e.g., Artificial Neural Networks, Linear Discriminant Analysis, regression models, Bayesian approaches, Decision Trees, and Random Forests.

*Limited Clinical Questions*. In this work we developed a predictive model able to address CN and sMCI subjects to a different therapeutic option with respect to pMCI and AD subjects. Our approach cannot be used for screening patients for specific AB or tau target drug clinical trials.

Approximately 27% of subjects meeting clinical inclusion criteria for mild-AD were found Ab-negative, thus, our multimodal classifier does not allow to avoid variance into analyses due to these patients. Aβ-negative mild-AD subjects are not expected to progress clinically on the expected trajectory, adding variance into analyses where a slowing of progression is being measured. Clinical trials of putative therapeutics for AD should use a baseline measure of brain Aβ or tau as an inclusion criterion, such as PET amyloid studies, even if a recent work demonstrated that measuring Aβ status from MRI scans in mild-AD subjects is possible and may be a useful screening tool in clinical trials (Tosun et al., 2016).

*Limited Neuropsychological Predictors*. Our work considered neuropsychological scores and sub-scores obtained from seven neuropsychological tests as candidate predictors. Whilst this offered a certain amount and details of information on different cognitive domains (a total of 64 scores were used as input data) as well as on behavioral and functional status, many other measures coming from other tests were excluded from our analysis only because not available for all the considered subjects. This limits our findings. A best accuracy in the prediction model could be achieved by using more neuropsychological measures (selected on the basis of their classification performance).

*Limited Dynamic View of the Disease Progression*. This study lacks of a dynamic view of the disease progression in terms of linking the imaging data between different time points. Although the different patterns of cerebral changes in AD/MCI over several time points have been compared in this paper, the proposed analysis was cross-sectional in nature at each time point, thus not investigating cross-time-point relationships with the predictive models. This would be a fundamental step for advancing our knowledge about neuropathological staging of Alzheimer-related changes. However, it should be kept in mind that in the last 10 years a plethora of longitudinal studies have provided consistent evidence on the evolution of neurodegenerative changes in AD brain. Recent advances in molecular neuroimaging have greatly facilitated our ability to detect neurodegenerative pathology *in vivo*, particularly in the very early stages of AD. As recently reviewed by Sperling et al. (2014), the inexorable progression of neurodegeneration characterizing patients with AD begins well more than a decade prior to the stage of clinically detectable symptoms. Amyloid-β (Aβ) accumulation may be evident 20 years before the stage of dementia, whilst substantial neuronal loss became evident by the stage of MCI. The challenge in this new era of neuroimaging application on AD is to demonstrate the real role played by the first hallmark of AD: Aβ accumulation. The general opinion is that Aβ is necessary, but not sufficient in isolation, to predict imminent decline along the AD trajectory. For this reason, structural neuroimaging can be useful for increasing the accuracy of automated diagnostic methods. Overall Aβ accumulation begins in the temporal cortex in very early AD phases, promoting dysmetabolism and neural losses. In the next phases, pathological changes move toward associative neocortex, mainly including orbitofrontal cortex, precuneus and prefrontal cortex, finally reaching the primary motor system along the AD trajectory. Our findings are thus in agreement with the well-known neurodegenerative staging of AD brain.

*Limited Prediction Over the Course of Disease*. In this study we were not able to establish if predicting progression to AD of MCI patients could be possible even at an earlier time than the *24 months* prior to the definite diagnosis, since the number of subjects provided by ADNI with an entire multimodal set of measures and with a longer follow up that 24 months is not sufficient for training-and-classification purposes.

Our classifier has been trained on measures of cognitive impairment obtained through clinically administered neuropsychological-test predictors. Thus, with this configuration, it cannot be used for screening presymptomatic subjects. However, in principle, our classifiers could be trained even over a different set of cognitive/behavioral and functional data, measured during daily life of CN subjects in order to capture domains that are affected first by the disease, eventually combined with their MRI brain studies in order to detect very subtle brain changes and on biological CSF with proper established cut points.

As pointed out in a recent review by ADNI (Weiner et al., 2017), longitudinal studies aimed at the early diagnosis and prognosis of AD are able to increase the power of clinical trials, as they can help in the selection of trial participants likely to decline. In these studies, the use of ML algorithms has been proved effective to measure surrogate diagnostic biomarkers, especially in challenges involving MCI subjects, but have been poorly validated for detecting the power of measures of longitudinal changes over time as surrogate predictive biomarkers of the disease.

In our study we demonstrated that it is possible to predict the conversion of MCI to probable AD up to 24 months before the definite diagnosis. Although better suited to trials of treatments aiming to repair brain tissue rather than clear Aβ, our approach may improve the feasibility of clinical trials by reducing costs and increasing the power to detect disease progression.

In conclusions, to our knowledge, this is one of the few works able to answer the question whether a multidisciplinary classification model coupling cognitive, functional and behavioral measures with structural MRI brain studies is better than a model based on structural MRIs alone. Since T1-weighted MRI scans are acquired routinely in clinical trials for other purposes and neuropsychological assessment can be easily performed to complement routine clinical trials, our multimodal pMCI classifier might be useful as a screening tool that could be applied to reduce the number of non-progressive subjects not to be treated.

## Author Contributions

CS, AC, and IC conceived, designed, and drafted this work. CS and IC performed the artificial-intelligence analysis. All authors critically revised, and approved the final version and agreed to be accountable for this work.

## Conflict of Interest Statement

The authors declare that the research was conducted in the absence of any commercial or financial relationships that could be construed as a potential conflict of interest.

## Footnotes

1 adni.loni.usc.edu

2 http://www.adni-info.org/Scientists/ADNIStudyProcedures.html
