# Supplementary material for: MRI Characterizes the Progressive Course of AD and Predicts Conversion to Alzheimer’s Dementia 24 Months Before Probable Diagnosis
Source: Front Aging Neurosci. 2018 May 24;10:135. doi: 10.3389/fnagi.2018.00135 (PMC5977985; doi:10.3389/fnagi.2018.00135)
Supplement: Supplementary file 1 [file Table_1.DOCX]

**Supplementary Materials**

**Table S1. List of neuropsychological scores and subscores used in this study.** The measures are indicated as reported in the ADNI data repository.

| FAQSOURCE  FAQFINAN  FAQFORM  FAQSHOP  FAQGAME  FAQBEVG  FAQMEAL  FAQEVENT  FAQTV  FAQREM  FAQTRAVL  FAQTOTAL  CLOCKCIRC  CLOCKSYM  CLOCKNUM  CLOCKHAND  CLOCKTIME  CLOCKSCOR  COPYCIRC  COPYSYM  COPYNUM  COPYHAND  COPYTIME  COPYSCOR  AVTOT1  AVERR1  AVTOT2  AVERR2  AVTOT3  AVERR3  AVTOT4  AVERR4  AVTOT5  AVERR5  AVTOT6  AVERR6  AVTOTB  AVERRB  DSPANFOR  DSPANFLTH  DSPANBAC  DSPANBLTH  CATANIMSC  CATANPERS  CATANINTR  CATVEGESC  CATVGPERS  CATVGINTR  TRAASCOR TRAAERRCOM  TRAAERROM  TRABSCOR TRABERRCOM TRABERROM  DIGITSCOR  BNTSPONT  BNTSTIM  BNTCSTIM  BNTPHON  BNTCPHON  BNTTOTAL  AVDEL30MIN  AVDELERR1  AVDELTOT  AVDELERR2 |
| --- |

**Table S2. Results in terms of p-values (multiple comparisons for one-way ANOVA) showing the statistical differences among the performance** **(accuracy) obtained at the four different time points.** Results are shown for all the possible binary combinations of time points and for the classification of *(CN + sMCI) vs (pMCI + AD)* using MRI features alone and PCA+FDR as feature-extraction-and-ranking technique.

|  | 24 months before stable diagnosis | 18 months before stable diagnosis | 12 months before stable diagnosis | Stable-diagnosis time point |
| --- | --- | --- | --- | --- |
| 24 months before stable diagnosis | - | - | - | - |
| 18 months before stable diagnosis | 0.71 | - | - | - |
| 12 months before stable diagnosis | 0.92 | 0.97 | - | - |
| Stable-diagnosis time point | 0.47 | 0.97 | 0.83 | - |

**Table S3. Results in terms of p-values (multiple comparisons for one-way ANOVA) showing the statistical differences among the performance (accuracy) obtained at the four different time points.** Results are shown for all the possible binary combinations of time points and for the classification of *(CN + sMCI) vs (pMCI + AD)* using MRI and neuropsychological features in combination and PCA+FDR as feature-extraction-and-ranking technique.

|  | 24 months before stable diagnosis | 18 months before stable diagnosis | 12 months before stable diagnosis | Stable-diagnosis time point |
| --- | --- | --- | --- | --- |
| 24 months before stable diagnosis | - | - | - | - |
| 18 months before stable diagnosis | 1 | - | - | - |
| 12 months before stable diagnosis | 0.98 | 0.95 | - | - |
| Stable-diagnosis time point | 0.27 | 0.22 | 0.46 | - |

**Table S4. Results in terms of p-values (multiple comparisons for one-way ANOVA) showing the statistical differences among the performance (accuracy) obtained at the four different time points.** Results are shown for all the possible binary combinations of time points and for the classification of *(CN + sMCI) vs (pMCI + AD)* using MRI features alone and PLS as feature-extraction technique.

|  | 24 months before stable diagnosis | 18 months before stable diagnosis | 12 months before stable diagnosis | Stable-diagnosis time point |
| --- | --- | --- | --- | --- |
| 24 months before stable diagnosis | - | - | - | - |
| 18 months before stable diagnosis | 0.85 | - | - | - |
| 12 months before stable diagnosis | 0.85 | 1 | - | - |
| Stable-diagnosis time point | 0.77 | 1 | 1 | - |

**Table S5. Results in terms of p-values (multiple comparisons for one-way ANOVA) showing the statistical differences among the performance (accuracy) obtained at the four different time points.** Results are shown for all the possible binary combinations of time points and for the classification of *(CN + sMCI) vs (pMCI + AD)* using MRI and neuropsychological features in combination and PLS as feature-extraction technique.

|  | 24 months before stable diagnosis | 18 months before stable diagnosis | 12 months before stable diagnosis | Stable-diagnosis time point |
| --- | --- | --- | --- | --- |
| 24 months before stable diagnosis | - | - | - | - |
| 18 months before stable diagnosis | 0.98 | - | - | - |
| 12 months before stable diagnosis | 0.94 | 1 | - | - |
| Stable-diagnosis time point | 0.87 | 0.98 | 1 | - |

**Table S6. Results in terms of p-values (paired-sample t-test) showing the pairwise statistical differences between the performance obtained using PCA+FDR vs those obtained using PLS, for accuracy (sensitivity/specificity).** Pairwise analysis were performed for each time point (columns) and for each domain (rows) separately.

|  | 24 months before stable diagnosis | 18 months  before stable diagnosis | 12 months  before stable diagnosis | Stable-diagnosis time point |
| --- | --- | --- | --- | --- |
| MRI | 0.03  (0.14/0.50) | 0.03  (0.30/0.23) | 0.06  (0.46/0.06) | 0.37  (0.83/0.21) |
| MRI +  Neuropsychological data | 0.27  (0.85/0.23) | 0.55  (0.43/1) | 0.58  (1/0.39) | 0.06  (0.37/0.01) |

**Table S7** **Best Neuropsychological predictors, corresponding status/domain/subdomain and ADNI-repository name of predictors found for the classification of (CN + sMCI) vs (pMCI + AD).** Results are reported for the three considered time points (i.e. *24 months before stable diagnosis*, *18 months before stable diagnosis*, and *12 months before stable diagnosis*) and for the time-zero point of stable diagnosis. Best neuropsychological predictors are sorted in descending order according to their frequency (the frequency of that measure in all loops). The status/domain/subdomain corresponding to the neuropsychological predictor is also reported. Neuropsychological scores and subscores are named as reported in the ADNI data repository.

| Time point | Neuropsychological predictor | Status/domain/  subdomain of predictor | ADNI-repository name of predictor |
| --- | --- | --- | --- |
| *24 months before stable diagnosis* | Partial Score of FAQ measuring the ability of the subject in remembering appointments, family occasions, holidays, medications.  Partial Score of FAQ measuring the ability of the subject in writing checks, paying bills, or balancing checkbook.  Partial Score of FAQ measuring the ability of the subject in assembling tax records, business affairs, or other papers.  Partial score of AVLT corresponding to the total of trial 5.  Partial Score of FAQ measuring the ability of the subject in keeping track of current events.  Partial score of AVLT corresponding to the total intrusions of trial 1.  Partial score of DS corresponding to the total correct answers in the Digit-Span-Backwards task.  Partial score of the Category Fluency test corresponding to the total correct answers in the Category-Fluency-Vegetables task.  Partial score of AVLT corresponding to the total correct answers after a 30-minutes delay. | Functional abilities  Functional abilities  Functional abilities  Memory and learning  Functional abilities  Memory and learning  Working memory  Language  Memory and learning | FAQREM  FAQFINAN  FAQFORM  AVTOT5  FAQEVENT  AVERR1  DSPANBAC  CATVEGESC  AVDEL30MIN |
| *18 months before stable diagnosis* | Partial Score of FAQ measuring the ability of the subject in writing checks, paying bills, or balancing checkbook.  Partial Score of FAQ measuring the ability of the subject in remembering appointments, family occasions, holidays, medications.  Partial score of AVLT corresponding to the total of trial 3.  Partial score of AVLT corresponding to the total of trial 5.  Partial score of AVLT corresponding to the total of trial 6.  Partial Score of FAQ measuring the ability of the subject in assembling tax records, business affairs, or other papers.  Partial score of FAQ measuring the ability of the subject in traveling out of the neighborhood, driving, or arranging to take public transportation.  Partial score of the CLOCK test, presence of the two hands.  Partial score of FAQ measuring the ability of the subject in shopping alone for clothes, household necessities, or groceries.  Partial Score of FAQ measuring the ability of the subject in keeping track of current events.  Total score of FAQ.  Partial score of AVLT corresponding to the total of trial 4.  Partial Score of BNT corresponding to the number of spontaneously given correct responses.  Partial score of BNT corresponding to the number of phonemic cues given to the subject.  Partial score of the CLOCK test corresponding to the symmetry of number placement.  Partial score of the CLOCK test corresponding to the presence of the two hands, set to ten after eleven.  Partial score of TMT corresponding to the time to complete Part A of the test.  Partial score of TMT corresponding to the time to complete Part B of the test.  Partial score of AVLT corresponding to the total correct answers after a 30-minutes delay.  Partial score of AVLT corresponding to recognition errors. | Functional abilities  Functional abilities  Memory and learning  Memory and learning  Memory and learning  Functional abilities  Functional abilities  Visuoconstructional reasoning  Functional abilities  Functional abilities  Functional abilities  Memory and learning  Language  Language  Visuoconstructional reasoning  Visuoconstructional reasoning  Complex attention  Complex attention  Memory and learning  Memory and learning | FAQFINAN  FAQREM  AVTOT3  AVTOT5  AVTOT6  FAQFORM  FAQTRAVL  CLOCKHAND  FAQSHOP  FAQEVENT  FAQTOTAL  AVTOT4  BNTSPONT  BNTPHON  CLOCKSYM  CLOCKTIME  TRAASCOR  TRABSCOR  AVDEL30MIN  AVDELERR2 |
| *12 months before stable diagnosis* | Partial Score of FAQ measuring the ability of the subject in writing checks, paying bills, or balancing checkbook.  Partial Score of FAQ measuring the ability of the subject in remembering appointments, family occasions, holidays, medications.  Partial score of AVLT corresponding to the total of trial 3.  Partial score of BNT corresponding to the number of correct responses following a phonemic cue.  Partial Score of FAQ measuring the ability of the subject in assembling tax records, business affairs, or other papers.  Partial score of FAQ measuring the ability of the subject in shopping alone for clothes, household necessities, or groceries.  Partial score of FAQ measuring the ability of the subject in traveling out of the neighborhood, driving, or arranging to take public transportation.  Total score of FAQ.  Partial score of AVLT corresponding to the total of trial 4.  Partial score of AVLT corresponding to the total of trial 5.  Partial score of AVLT corresponding to the total correct answers after a 30-minutes delay.  Partial score of AVLT corresponding to the total of trial 6.  Partial Score of FAQ measuring the ability of the subject in keeping track of current events.  Partial Score of FAQ measuring the ability of the subject in paying attention to and understanding a TV program, book, or magazine.  Total score of the CLOCK test. | Functional abilities  Functional abilities  Memory and learning  Language  Functional abilities  Functional abilities  Functional abilities  Functional abilities  Memory and learning  Memory and learning  Memory and learning  Memory and learning  Functional abilities  Functional abilities  Visuoconstructional reasoning | FAQFINAN  FAQREM  AVTOT3  BNTCPHON  FAQFORM  FAQSHOP  FAQTRAVL  FAQTOTAL  AVTOT4  AVTOT5  AVDEL30MIN  AVTOT6  FAQEVENT  FAQTV  COPYSCOR |
| *Time-zero point of stable diagnosis* | Partial Score of FAQ measuring the ability of the subject in writing checks, paying bills, or balancing checkbook.  Total score of FAQ.  Partial score of AVLT corresponding to the total of trial 4.  Partial Score of FAQ measuring the ability of the subject in remembering appointments, family occasions, holidays, medications.  Partial Score of FAQ measuring the ability of the subject in paying attention to and understanding a TV program, book, or magazine.  Partial score of FAQ measuring the ability of the subject in traveling out of the neighborhood, driving, or arranging to take public transportation.  Partial Score of FAQ measuring the ability of the subject in assembling tax records, business affairs, or other papers.  Partial score of FAQ measuring the ability of the subject in preparing a balanced meal.  Partial score of AVLT corresponding to the total of trial 6.  Partial score of FAQ measuring the ability of the subject in playing a game of skill such as bridge or chess, working on a hobby.  Partial score of AVLT corresponding to the total correct answers after a 30-minutes delay. | Functional abilities  Functional abilities  Memory and learning  Functional abilities  Functional abilities  Functional abilities  Functional abilities  Functional abilities  Memory and learning  Functional abilities  Memory and learning | FAQFINAN  FAQTOTAL  AVTOT4  FAQREM  FAQTV  FAQTRAVL  FAQFORM  FAQMEAL  AVTOT6  FAQGAME  AVDEL30MIN |
